# A neurobiology of learning beyond the declarative non-declarative distinction

**DOI:** 10.3389/fnbeh.2013.00161

**Published:** 2013-11-14

**Authors:** Daniele Ortu, Manish Vaidya

**Affiliations:** Neurobehavioral Laboratory, Department of Behavior Analysis, University of North TexasDenton, TX, USA

**Keywords:** stimulus-stimulus relations, stimulus-response relations, reinforcement, hippocampus, basal ganglia, declarative learning, consciousness

## Introduction

In neuroscience and psychology it has become common to distinguish between declarative and non-declarative forms of learning (e.g., Squire and Zola, 1996). The perspective (Squire, 2004, p. 173) that declarative learning “refers to the capacity for conscious recollection about facts and events and is the kind of memory that is impaired in amnesia and dependent on structures in the medial temporal lobe” developed when it was observed that patients with hippocampal damage were able to learn and improve on “procedural” tasks, such as hand-eye coordination, without being able to remember the specific learning episodes (e.g., Scoville and Milner, 1957; Squire, 1992). Such evidence led researchers (e.g., Moscovitch, 1995) to suggest that there are two separate learning systems: one involved in declarative, conscious, learning situated in the Medial Temporal Lobe (MTL) comprising the hippocampus, and another involved in non-declarative, implicit, skill/habit learning involving the basal ganglia.

An interpretation of the roles of the hippocampus and the basal ganglia in learning independent from the declarative/non-declarative dichotomy may allow understanding the role of neural structures critical in learning without relying on phenomenological categories—difficult to examine from a scientific perspective—such as awareness and consciousness. The very nature of what consciousness is in fact still hotly debated (e.g., Morin, 2006) and grounding an entire taxonomy of learning and memory on consciousness-based criteria might complicate, instead of simplifying, scientific interpretations, and progress. An alternative perspective to the declarative—non-declarative distinction (e.g., Packard and McGaugh, 1992; McDonald and White, 1993) emphasizes the role of the hippocampus in learning stimulus-stimulus relations, and of the dorsal striatum in acquiring stimulus-response relations. Such perspective is consistent with a recent review (Henke, 2010) suggesting that neurobiological models of learning based on consciousness might not be adequate to describe the available experimental evidence.

## The hippocampus and stimulus-stimulus relations

The interpretation that the hippocampus is involved in learning arbitrary stimulus-stimulus relations is supported by the finding that amnestic patients with MTL lesions are impaired in associative recognition procedures compared to item recognition (Giovanello et al., 2003). Similarly, Henke et al. (1997) described hippocampal and parahippocampal activation during associative learning but not during item learning. Moreover recollecting behavior, defined by neuroscientists and psychologists in terms of the ability to describe the unique contextual details about a remembered episode (e.g., Wilding, 2000), and considered to be an example of rapid learning of arbitrary stimulus-stimulus relationships, is also typically regarded to be hippocampus-dependent (e.g., Yonelinas et al., 2005). The mnemonic deficits observed in hippocampal patients may therefore, be described as “losing the ability to learn unique stimulus-stimulus configurations,” including what has been labeled as “episodic recollection,” as opposed to “losing declarative memory.” The literature on episodic learning is in fact grounded on the idea that episodic learning is declarative by definition (e.g., Eichenbaum, 2000), but by redefining episodic learning as rapid acquisition of a variety of arbitrary stimulus-stimulus configurations—both in the time and space domain—the “problem” of consciousness becomes less relevant.

Is there experimental evidence that the hippocampus is involved in learning stimulus-stimulus associations regardless of “consciousness”? The idea that hippocampus-mediated learning is independent from degrees of consciousness is supported by experiments showing that the hippocampus is active during stimulus-stimulus learning even when stimulation occurs subliminally (i.e., when the stimulus duration is very short—e.g., 17 ms—not allowing participants to discriminate stimulus presentation; Henke et al., 2003). This finding goes directly against the interpretation that the hippocampus is “specialized” in declarative learning, suggesting instead that its function in learning is independent from the participant's “awareness.” Further considerations inconsistent with the idea that learning with awareness and learning without awareness constitute separate processes come from the large amount of evidence showing strengthening by reinforcement of verbal responses (e.g., Gupta, 1976, 1990; Williams, 1977; Gupta and Nagpal, 1978; Nagpal and Gupta, 1979; Gupta and Shukla, 1989; Jolliffe and Nicholas, 2004). If language-mediated learning is by definition declarative and dissociable from skill/habit learning—such as selection by reinforcement—why is then language sensitive to reinforcement?

To answer this question it may be useful to consider an interpretation (Donahoe and Palmer, 1994) suggesting that if the hippocampus is involved in selecting arbitrary stimulus-stimulus relationships, it then should play an important role in the acquisition of verbal responses because verbal learning (from a stimulus equivalence perspective; Sidman, 1994) implies arbitrary relations and interchangeability among several (e.g., written, auditory, and visual) forms of stimulation. In other words, verbal responses imply arbitrary stimulus-stimulus associations. If this is true, then it is to be expected that verbal learning (typically described as a form of explicit learning) cannot take place without the hippocampus, which is what happened to Henry Molaison (Scoville and Milner, 1957) and other hippocampal amnestic patients. Hippocampal organisms are consequently expected to display deficits in tests that rely on strengthening arbitrary stimulus-stimulus relations *including verbal ones*. Verbal learning, however, according to this interpretation, does not have a special status compared to non-verbal learning. Verbal behavior may then be sensitive to reinforcement because MTL function is dependent on neuromodulation (e.g., Gaffan, 2002) and the hippocampus receives a specific input to area CA1 from the Ventral Tegmental Area (Gasbarri et al., 1994; Martig and Mizumori, 2010), a region specifically sensitive to reinforcing stimulation.

## The basal ganglia and stimulus-response relations

When reinterpreting the role of the basal ganglia in learning without using the category of consciousness, a description of their involvement in reinforcement learning mechanisms may provide a useful, “phenomenology-free,” alternative. The basal ganglia have in fact been previously described (e.g., White, 1997) as involved in reinforcement-mediated acquisition of stimulus-response relations. For example, experiments have shown that dorsal striatum lesions impair a rat's performance in a win-stay task in a radial maze, in which the rat needs to learn to approach lit maze arms (McDonald and White, 1993). The light illuminating a specific arm of the maze acquires therefore, a discriminative function for approaching behavior, and such acquisition is critically dependent on the dorsal striatum. Recent evidence (Featherstone and McDonald, 2004) has moreover shown that the dorsolateral striatum—but not the dorsomedial striatum—appears to be critically involved in stimulus-response learning mediated by reinforcement. Modulation by reinforcement is possible because the basal ganglia are innervated by dopaminergic pathways originating from the Substantia Nigra and the Ventral Tegmental Area (e.g., Graybiel, 1990) and presentation of reinforcers determines a release of dopamine thought to strengthen synaptic connections between neurons co-active at the moment of reinforcement (e.g., Stein and Belluzzi, 1988, 1989). The dopaminergic input to the dorsal striatum activated at the moment of reinforcement is therefore, according to this account, responsible for the selection of a specific stimulus-response relationship (i.e., activation of a similar sensory response in the future will increase the likelihood of a similar motor response).

## Common sensitivity to consequences

Interpretations of the roles in learning of the hippocampus and the basal ganglia that do not rely on the declarative non-declarative distinction have therefore, already a clear role in the literature. Moreover, recent evidence suggests that consequence based learning—also described as feedback learning—historically considered to be part of a non-declarative learning system mediated by the basal ganglia and specifically the striatum, is also mediated by the hippocampus (Foerde et al., 2013; see also Ortu et al., 2013). Foerde et al. specifically demonstrated experimentally how feedback learning—when feedback is delayed (i.e., 7 s)—is dependent on the hippocampus but not on the striatum. Relatedly, Gaffan (2002) has described how the excision of neuromodulatory pathways afferent to temporal areas leads to a dense form of amnesia, suggesting that environmental feedback constitutes a necessary requirement for MTL areas to function. Since such neuromodulatory pathways carry information about the reinforcing/punishing/novelty value of current environmental and proprioceptive stimulation, it seems overall improbable that “learning by observation” can occur without constant feedback to areas involved in learning about the “value” of incoming stimulation. It appears therefore, that both basal ganglia and hippocampus mediated learning are dependent on “environmental” learning signals, i.e., both are involved in forms of feedback learning, conditional on the stimulation incoming from currently experienced environments.

## Further observations

Squire(1992, p. 210) has proposed that non-declarative learning involves behavioral change while declarative learning involves recollection: “Whereas declarative memory concerns recollection, non-declarative memory concerns behavioral change. In non-declarative memory, information is acquired as changes within specific perceptual or response systems, independently of memory for the prior encounters that led to behavioral change.” The proposal that declarative learning concerns recollection and non-declarative learning concerns behavioral change is problematic because it implies that recollection/remembering does *not* involve behavioral change. The literature focusing on recognition learning, however, suggests quite the opposite: the occurrence of recollection in recognition experiments is typically validated by reliable behavioral change in source accuracy tasks (e.g., Wilding, 2000). We know with reasonable confidence that participants are remembering only when they are able to describe contextual information relative to a specific studied episode. The behavioral measure therefore, validates the neural correlate of remembering. For example, in the case of Event Related Potentials (ERPs), the Left Parietal Effect—considered to index recollecting behavior—is validated by behavioral results in source accuracy tasks as its magnitude increases with higher source accuracy scores (Wilding, 2000).

## Limitations

As the current debate about theoretical frameworks for characterizing neural structures involved in learning progresses, it is essential to highlight some of the weaknesses of the perspective presented here. One limitation of the fMRI evidence described by Henke et al. (2003), showing that the hippocampus is active during stimulus-stimulus learning when stimulation is presented subliminally, is that the detection of hippocampal activation during subliminal associative learning does not rule out the possibility of an interaction among different brain areas. The MTL structure may in fact not be solely responsible for those instances of stimulus-stimulus learning, and by implication its activation may constitute a necessary but not sufficient prerequisite. Consistently, there are cases of statistical learning, often considered to be a case of stimulus-stimulus learning, assessed in patients with MTL lesions (e.g., Nissen and Bullemer, 1987). Overall, it appears therefore, probable that the hippocampus acts in conjunction with other areas during stimulus-stimulus learning. Similarly, with regards to stimulus-response learning, even if the basal ganglia play a crucial role in the selection of stimulus-response relations it is important to note that other structures such as the cerebellum and the amygdala have also been implicated in this form of learning (e.g., Baxter and Murray, 2002; Boyden et al., 2004) and their role may be necessary for stimulus-response learning to occur.

## Conclusion

The traditional taxonomy that distinguishes between neural systems supporting declarative and non-declarative forms of learning may be inadequate, as experimental and theoretical work suggests that other criteria may be more useful in categorizing the role of neural structures involved in learning such as the hippocampus and the basal ganglia. The hippocampus, traditionally considered to be the core of a declarative learning system, can in fact be described as involved in strengthening stimulus-stimulus relations—even when stimuli are presented subliminally. The basal ganglia, historically described as fundamental in a non-declarative learning system involved in feedback based skill/habit learning, may similarly be better understood as critical in stimulus-response learning, regardless of awareness or consciousness. Moreover, both structures are sensitive to neuromodulation activated by environmental or proprioceptive stimulation. Clear mechanisms describing the role of the basal ganglia in stimulus-response binding and the hippocampus in stimulus-stimulus binding have been described and are entirely independent from classifications based on awareness and consciousness. Overall, these interpretations offer a “phenomenology free” account of the involvement of brain structures in learning and—by taking explicitly into account how environmental changes trigger neuromodulation involved in selecting stimulus-stimulus and stimulus-response relations—may also provide a selectionist framework to understand neurobehavioral adaptations.
